# Family of graphene-assisted resonant surface optical excitations for terahertz devices

**DOI:** 10.1038/srep35467

**Published:** 2016-10-14

**Authors:** I-Tan Lin, Jia-Ming Liu, Hsin-Cheng Tsai, Kaung-Hsiung Wu, Jheng-Yuan Syu, Ching-Yuan Su

**Affiliations:** 1 Electrical Engineering Department, University of California, Los Angeles, Los Angeles, California 90095, USA; 2 Department of Electrophysics, National Chiao-Tung University, Hsinchu, Taiwan; 3 Graduate Institute of Energy Engineering, National Central University, Taoyuan, Taiwan

## Abstract

The majority of the proposed graphene-based THz devices consist of a metamaterial that can optically interact with graphene. This coupled graphene-metamaterial system gives rise to a family of resonant modes such as the surface plasmon polariton (SPP) modes of graphene, the geometrically induced SPPs, also known as the spoof SPP modes, and the Fabry-Perot (FP) modes. In the literature, these modes are usually considered separately as if each could only exist in one structure. By contrast, in this paper, we show that even in a simple metamaterial structure such as a one-dimensional (1D) metallic slit grating, these modes all exist and can potentially interact with each other. A graphene SPP-based THz device is also fabricated and measured. Despite the high scattering rate, the effective SPP resonances can still be observed and show a consistent trend between the effective frequency and the grating period, as predicted by the theory. We also find that the excitation of the graphene SPP mode is most efficient in the terahertz spectral region due to the Drude conductivity of graphene in this spectral region.

The graphene-metamaterial hybrid devices studied in the literature have a structure similar to the one shown in Fig. 1a. The miniature structure on the metamaterial substrate is usually periodic. Some of the patterns studied in the literature are shown in the insets of Fig. 1a; from left to right are 1D grating^1^,^2^, two-dimensional (2D) holes^3^,^4^, rings^5^, and split ring resonators (SRR)^6^,^7^,^8^. Depending on the design and the physical parameters, different surface excitations are excited. One such excitation is the spoof SPP modes^9^. The resonance frequency *v* of a spoof SPP mode is determined by the design parameters of the structure. For normally incident radiation of a wavelength *λ* that is much larger than the structural dimensions of the metamaterial patterns, the resonance frequencies of 1D grooves with *h* < *d*_1_, 1D slits with *h* = *d*_1_, 2D holes, and 2D rings, as shown in the insets of Fig. 1a, are given approximately by *v* ≈ *c*/4*n*_eff_*d*_1_^3^, *v* ≈ *c*/2*n*_eff_*d*_1_^4^, *v* ≈ *c*/2*n*_eff_*w*^9^, *v* ≈ *c*/2*πrn*_eff_^5^, respectively, where *n*_eff_ is the effective index of the mode and *r* is the radius of the ring. If the ring is cut so that the symmetry is broken, the resulting SRR structure can be regarded as an LC circuit with a new resonance frequency of 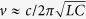. Taking 1D grating as an example, the characteristic transmittance is shown in the inset of Fig. 1b for the excitation of the spoof SPPs. By tuning the chemical potential *μ*, we effectively change the boundary condition on one side of the gap, and thus the excitation can be either suppressed or enhanced. If a graphene SPP mode is also excited, there can be a coupling of the graphene SPP mode and the spoof SPP mode. As a result, the spoof SPP mode is split into two coupled modes, as can be seen in the inset of Fig. 1b for two transmittance peaks. One major issue of the devices based on the excitation of spoof SPPs is that the physical size of the pattern has to be large so that the resonance frequency can be in the THz spectral region. The typical lengths of the relevant structural parameters are in the range of 50 to 100 μm for the resonance frequency in the sub-THz region^5^,^7^. Furthermore, the resonance frequency is fixed and cannot be tuned by adjusting the chemical potential of graphene unless the spoof SPP mode is coupled with other quasiparticles.

Another possible excitation for the device shown in Fig. 1a is the FP modes, as shown in Fig. 1c. When the graphene sheet is placed sufficiently far away from the metallic grating, FP modes are supported between the graphene sheet and the metallic grating. By moving the Fermi level away from the Dirac point, the graphene sheet changes from weakly conducting to highly conducting. As a result, the boundary condition on the graphene side of the FP cavity is modified, and the corresponding resonance frequency of the FP mode changes accordingly, as shown in the inset of Fig. 1c. Again, the issue of this type of devices is the large footprint. For a resonance frequency in the sub-THz region, the thickness of the substrate is around 100 μm to few hundred micrometers^10^,^11^. The operational range is also limited even with a large doping of the graphene sheet.

For the periodic structure shown in Fig. 1a, it is also possible to excite graphene SPPs, as shown in Fig. 1d. By increasing the chemical potential, the electron density and the conductance increase, and the resonance frequency of a graphene SPP mode, corresponding to the transmittance trough, is blue-shifted, as shown in the inset of Fig. 1d where the chemical potential *μ*_2_ > *μ*_1_. Therefore, by adjusting the chemical potential of graphene, the resonance frequency can be tuned and thus the transmitted signal can be switched on and off. Compared to the devices based on other types of surface excitation, the device shown in Fig. 1d has the smallest footprint because of the strong confinement of the graphene SPPs. This type of devices also benefits from the great tunability of the resonance frequency through electrostatic doping^2^. For a scattering rate of 1 ps^−1^, the modulation depth is around 90% at 0.6 THz, as shown in the inset of Fig. 1d. This value is larger than that of devices based on Fig. 1c^11^,^12^, and much larger than the experimentally measured values, which are 15% or below, for devices based on SRR or monolayer graphene on a flat substrate^10^,^13^,^14^. In this paper, we mainly focus on the devices based on the excitation of graphene SPPs.

## Results

### Numerical simulation

**Figure 1:**
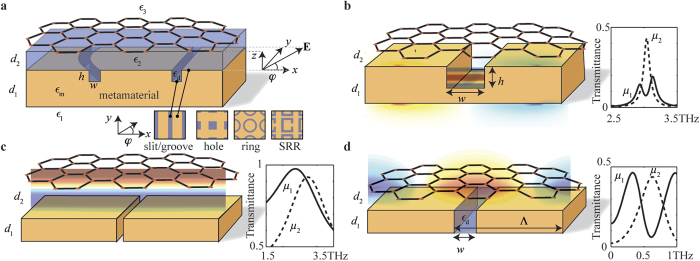
Various excitations of graphene-based THz devices. (**a**) Sketch of a graphene-metamaterial structure. The miniature structure of the metamaterial can take the form of periodic slits, holes, rings, or SRR, as shown in the insets. By coupling graphene with a metamaterial surface, (**b**) a spoof SPP mode, (**c**) a FP mode, or (**d**) a graphene SPP mode can be excited by normally incident light. For all figures, 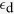, 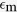, 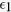, 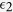, and 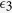 are the permittivities of the gap filling, the metamaterial, the medium below the metamaterial, the medium below graphene, and the medium above graphene, respectively; *d*_1_ is the thickness of the metamaterial; *d*_2_ is the distance between the graphene sheet and the metamaterial. In the case of a 1D grating structure being the metamaterial structure as shown in (**b**–**d**) Λ is the period, and the gap is characterized by a width of *w* and a height of *h*. The representative transmittance curves as a result of the excitation of spoof SPP, FP modes, and graphene SPP are shown in the insets in (**b**–**d**) respectively, calculated using the classical modal method^15^. The parameters are *h* = *d*_1_, *w* = 0.1 μm, Λ = 3 μm, chemical potential *μ*_1_ = 100 meV, polarization angle *ϕ* = 0°, scattering rate *γ* = 1ps^−1^ for the graphene sheet^23^, 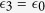, and 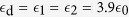, where 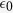 is the permittivity of free space. The Drude model is used for the graphene sheet, and the metal is assumed to be a perfect conductor. The increased chemical potentials used for the insets in (**b**–**d**) are *μ*_2_ = 200 meV, 400 meV, and 350 meV, respectively. In (**b)** d_1_ = 25 μm, d_2_ = 10 nm. In (**c**) d_1_ = 10 nm, d_2_ = 25 μm. In (**d)** *d*_1_ = *d*_2_ = 10 nm.

**Figure 2:**
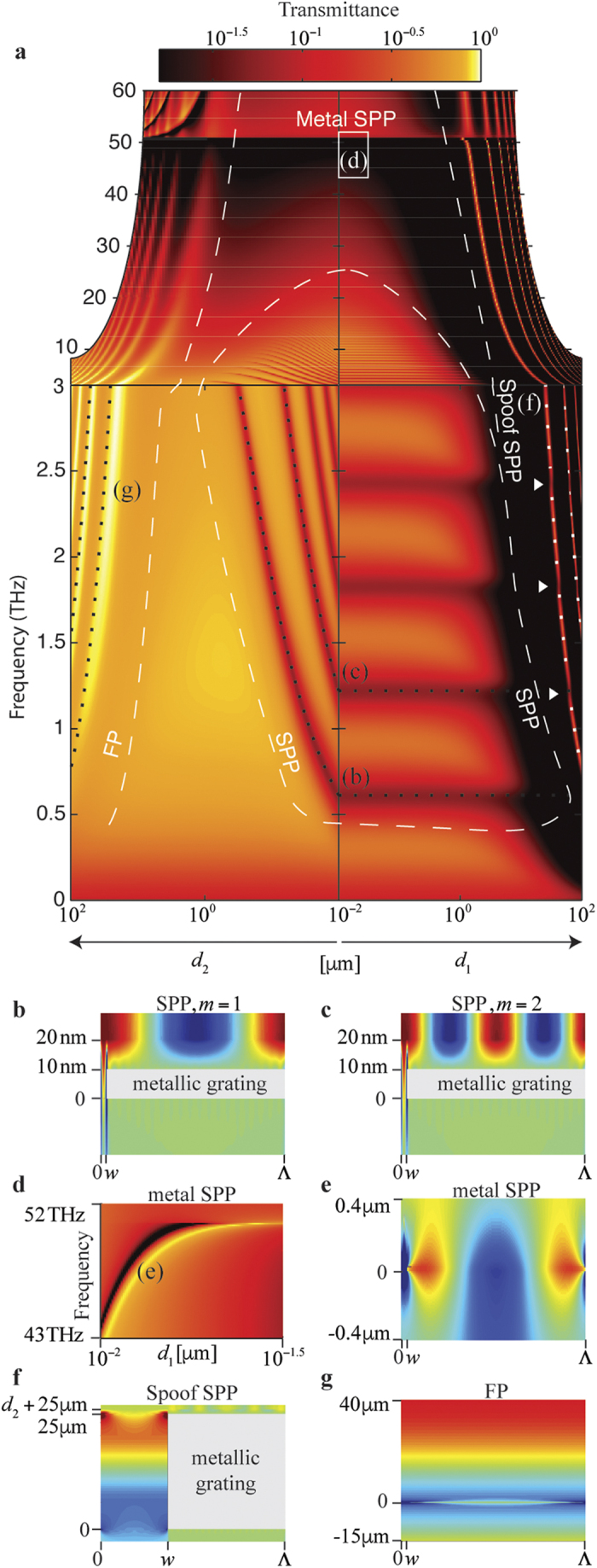
Dispersion map of metal SPP, graphene SPP, spoof SPP, and FP modes. (**a**) Transmittance as a function of *d*_1_ and *d*_2_ calculated using the classical modal method^15^. Other physical parameters used for the simulation are the same as those used for the insets in Fig. 1(b–d). Areas of the dominant modes are separated approximately by the white dashed curves. The dotted curves for the first two orders of graphene SPP modes and the spoof SPP modes are obtained from Eqs (1) and (2), respectively. The dotted curves for the FP modes are given by *v* = *mc*/2*n*_2_*d*_2_, where *m* is a positive integer. The dispersion curves of the high-order FP and spoof SPP modes are not plotted as the linewidth become exponentially thin below the resolution of the figure. (**b**,**c**) The electric field *E*_*x*_ of the graphene SPP modes for *d*_1_ = *d*_2_ = 10 nm. The frequencies are *v* = 0.6 THz and 1.2 THz labeled (b, c) in (**a**), respectively. (**d**) The metal SPP dispersion curve for the region marked by the white box labeled (d) in (**a**) assuming 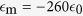. (**e**) Field distribution of *E*_*x*_ for the parameters of *d*_1_ = 12.6 nm and *v* = 47 THz marked by (e) in (**d**).(**f**), Field distribution of *E*_*x*_ for the parameters of *d*_1_ = 25 μm and *v* = 2.9 THz labeled (f) in (**a**). The structure shown is not to scale. (**g**) Field distribution of *E*_*x*_ for the parameters of *d*_2_ = 25 μm and *v* = 2.4 THz labeled (g) in (**a**). The metallic grating is not discernible in (**e**) and (**g**) because of its small thickness *d*_1_ compared to the dimensions of the graphs.

The dispersion curves of graphene SPP, spoof SPP, and FP modes can be found by locating the resonance frequency in the transmittance spectrum. For normally incident light, the dispersion map calculated using the modal method^15^ are plotted in Fig. 2 as a function of *d*_1_ (right panel) and *d*_2_ (left panel). The parameters are the same as those used for the insets in Fig. 1b–d. Three regions are clearly seen where graphene SPP, spoof SPP, and FP modes are respectively dominant. In the case of *w* ≪ Λ, the resonance frequency *ω* of the *m*th-order SPP mode is given by^16^


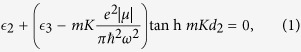


where *K* = 2*π*/Λ is the wave number of the periodic metallic grating that has a period of Λ. In Eq. (1), the Drude model for graphene is used, and the losses assumed to be negligible (see Supplementary Information); the metal is assumed to be a perfect conductor, which is a good approximation in the THz spectral region. The dispersion curves of graphene SPP modes solved by using Eq. (1) are plotted in Fig. 2a; the dotted curves labeled (b) and (c) are for the first- and second-order graphene SPP modes. These curves follow the transmittance troughs that correspond to the location of the SPP excitation. The fields of these graphene SPP modes are confined on the graphene sheet, as shown in Fig. 2b,c for the first and second orders, respectively. Above 10 THz in Fig. 2a, the dispersion curves are no longer observable because the Drude conductivity of graphene that supports these SPP modes drops significantly in the high frequency region.

As the optical frequency increases, the excitation of the metal SPP mode becomes possible. In the case of 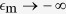 for metal in the THz spectral region, the metal SPP mode satisfies the Rayleigh condition 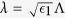
4, which gives a transmittance trough at 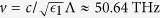 in Fig. 2a. For a finite but large 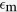, for example 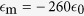, the coupling of the two surfaces of metallic grating becomes possible for a small thickness *d*_1_; then, the dispersion curve is altered as shown in Fig. 2d for the location marked by a white box labeled (d) in Fig. 2a. A sharp resonance characterized by a transmittance maximum followed by a transmission minimum as shown in Fig. 2d is a characteristic feature of a Fano resonance supported by a metallic grating^17^. This Fano resonance is the result of the interference between the discrete metal SPP mode and the non-resonant continuum^18^. A representative electric field distribution of the metal SPP mode is plotted in Fig. 2e.

As the thickness *d*_1_ of the grating increases, the excitation of spoof SPP modes in THz spectral region becomes possible. We consider the first-order grating because the effect of the high-order gratings is hardly observable in the transmission spectrum^3^,^4^. For a first-order grating, the dispersion of the spoof SPP modes in the long-wavelength limit of *λ* ≫ Λ and *λ* ≫ *w* is given by^4^


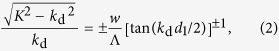


where *k*_d_ = *n*_d_*ω*/*c* and 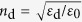. Because of the periodicity of the tangent functions, Eq. (2) gives two sets of solutions when plus and minus signs are taken respectively. When *K* ≫ *k*_d_, the left-hand side of Eq. (2) approaches infinity, and thus the solution is found by requiring the argument *k*_d_*d*_1_/2 on the right-hand side of Eq. (2) to be multiples of *π*/2, which gives *v* = *mc*/2*n*_d_*d*_1_ for the resonance frequency of the *m*th-order mode. The first two modes are plotted as dotted curves labeled (f) as a group, which agree well with the simulation results in the right panel of Fig. 2a. In the low-frequency region where the coupling with the metal SPP mode is negligible, the field is confined inside the gap, as shown in Fig. 2f. In this case, a spoof SPP mode is also called a cavity mode, which plays an important role in extraordinary optical transmission (EOT)^17^,^19^. Spoof SPP modes can also couple with graphene SPP modes to open up gaps in the dispersion curves, as indicated by the white arrows in Fig. 2a. The location of the gaps can be tuned by adjusting the chemical potential of graphene; thus the transmittance of the graphene-metamaterial structure can be tuned accordingly, as shown in Fig. 1b.

When the wavelength of light is comparable to the distance *d*_2_, a FP cavity is formed between the graphene and the metallic grating with the boundary conditions determined by the chemical potential of graphene. The *m*th-order resonance frequency of a FP cavity characterized by a dielectric constant *n*_2_ and a thickness *d*_2_ is given by *v* = *mc*/2*n*_2_*d*_2_, where *m* is a positive integer. The first two modes are plotted as dotted curves labeled (g) as a group in the left panel of Fig. 2a. The curves approximately agree with the simulation results. The discrepancy is due to the finite conductance of graphene and the unbalanced dielectric indices *n*_1_ and *n*_3_ that make the FP cavity imperfect. The transmittance is tuned by adjusting the chemical potential of graphene to achieve the functionality of the device shown in Fig. 1c.

### Coupling among modes

**Figure 3:**
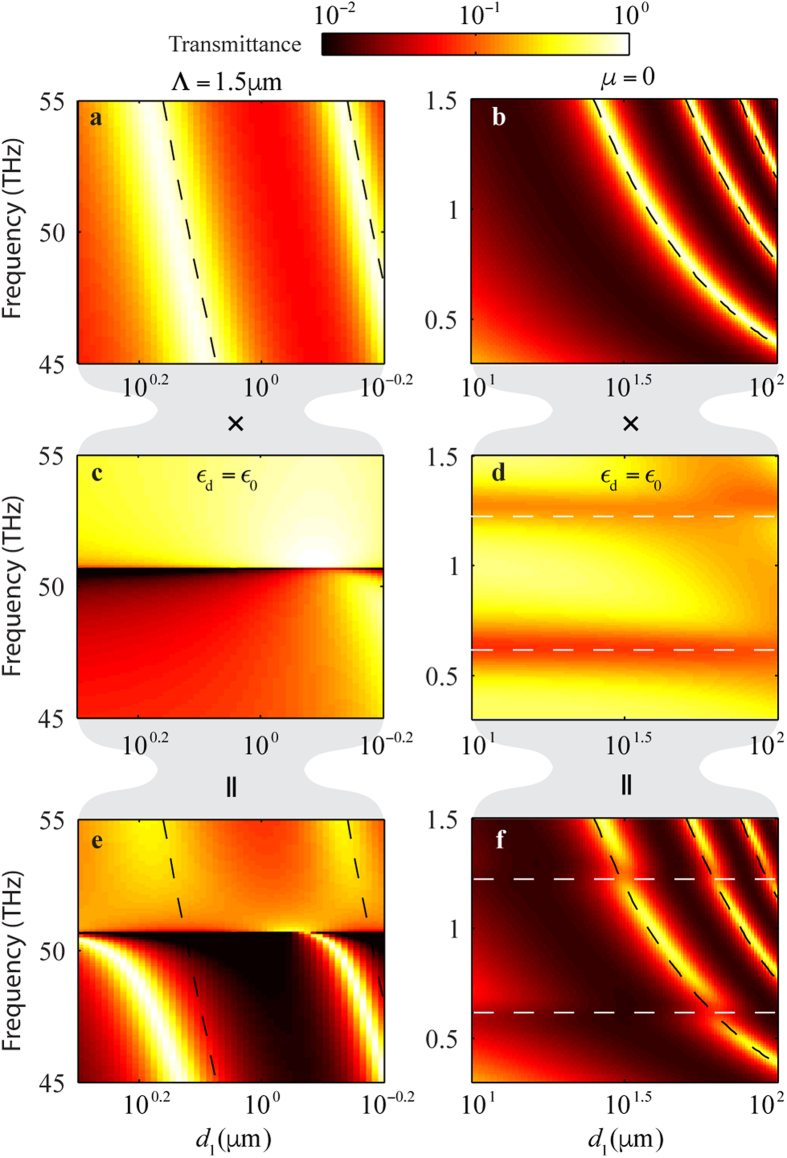
Coupling of the cavity modes with the metal and graphene SPP modes. Unless otherwise specified in the figures, the simulation parameters used are the same as those for Fig. 2a, except for *w* = 0.5 μm and 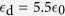. Black and white dashed curves represent the dispersion curves of the cavity modes and the graphene SPP modes, respectively. (**a**,**b**) Transmittance map in the presence of the cavity modes. (**c**,**d**) Transmittance map in the presence of the metal SPP and graphene SPP modes, respectively. (**e**) Transmittance map in the presence of the coupling between the cavity modes and the metal SPP mode. (**f**) Transmittance map in the presence of the coupling between the cavity modes and the graphene SPP modes.

For the completeness and the later discussion of graphene SPP coupling, we first discuss the coupling between the metal SPP mode and the cavity modes. As discussed in the preceding section, a spoof SPP mode that is far away from the metal SPP dispersion curve is essentially a cavity mode with its energy confined within the gaps. As the dispersion curve of the cavity modes approaches the resonance frequency of a metal SPP mode, the resultant spoof SPP mode becomes a mixed state of metal SPP and spoof SPP, with the field distribution in the gaps and also on the metal grating. The dispersion curves of two spoof SPPs, characterized by a high transmittance^17^, are shown in Fig. 3e. Unless otherwise specified, the simulation parameters used for Fig. 3 are the same as those for Fig. 2a, except for *w* = 0.5 μm and 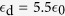. As we shall see later, these values are chosen so that the coupling effects become more discernible as the dispersion curves of the spoof SPP modes move toward the low-*d*_1_ region where graphene SPPs dominate. As can be seen in Fig. 3e, the dispersion curves bend toward the dispersion curve of the metal SPP mode; thus, they can no longer be described by the dispersion characteristics of the cavity modes, given by *v* = *mc*/2*n*_d_*d*_1_, which are plotted as the dashed curves. To clearly see the dispersion curves of the cavity modes and the metal SPP mode in this coupling region, we tune the structural parameters so that the excitation of either the cavity modes or the metal SPP mode is suppressed. In Fig. 3a, the dispersion curve of the metal SPP mode is eliminated by halving the period so that the resonance frequency of metal SPP is lifted. As can be seen, without the presence of the metal SPP, the spoof SPP mode is well described by the cavity mode, which has a dispersion curve given by *v* = *mc*/2*n*_d_*d*_1_. In Fig. 3c, the dispersion curves of the cavity modes are eliminated by setting 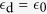 so that the resonance frequencies of the cavity modes shift toward to the high-*d*_1_ region. As can be seen, the dispersion curve of the metal SPP mode is characterized by a low transmittance and a weak dependence on *d*_1_. For a metal of a finite conductivity, the resonance frequency redshifts away from the Rayleigh condition of 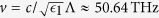
17,18,19.

A similar study can be performed on the coupling between the graphene SPP modes and the cavity modes. The coupling of the graphene SPP and the cavity modes are shown in Fig. 3f. The dashed curves represent the dispersion curves of graphene SPPs given by (1) and the cavity modes given by *v* = *mc*/2*n*_d_*d*_1_ in the absence of the coupling effect. Similar to the case of metal plasmons, the dispersion curves of graphene SPPs are characterized by the low transmittance. This low transmittance effectively suppresses the transmittance at the intersections of the dispersion curves and thus creates multiple bands in the dispersion curves. This gap opening is also shown in Fig. 2a, and in the inset of Fig. 1b. Although not as strong as in the case of metal plasmon coupling, the dispersion curves of the cavity modes also deviate from those of the decoupled cavity modes and slightly bend toward the dispersion curves of graphene SPPs. To clearly see the dispersion curves of the cavity modes and the graphene SPP modes in this coupling region, we tune the structural parameters so that the excitation of either the cavity modes or the graphene SPP modes are suppressed. For example, by reducing the chemical potential *μ* to zero, there is no graphene SPPs, and the aforementioned gap openings are closed, as shown in Fig. 3b. Alternatively, by setting 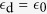 so that the resonance of the cavity modes is “turned off”, there are only the SPP modes in the plotting region, as shown in Fig. 3d. By comparing Fig. 3d,f, it is apparent that the gap opening happens at the resonance frequency of graphene SPPs.

### Scattering effect

**Figure 4:**
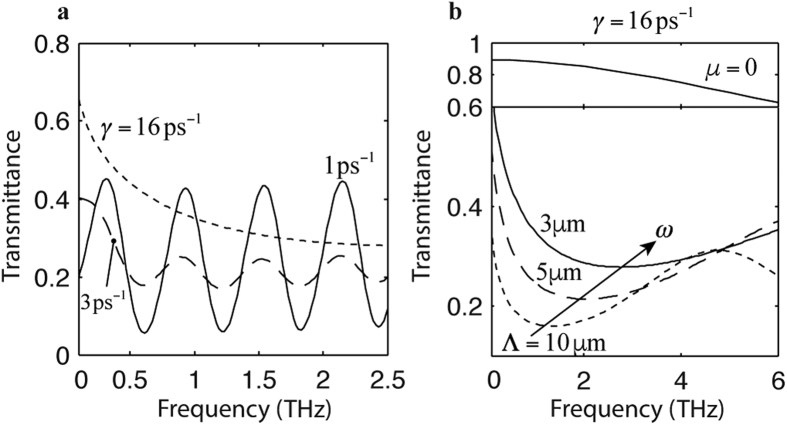
Scattering effect on the transmittance curves. Unless otherwise specified in the figures, the simulation parameters used are the same as those for Fig. 2a with *d*_1_ = *d*_2_ = 10 nm. (**a**) Transmittance of graphene on the grating with the scattering rates of *γ* = 1 ps^−1^(solid curve), 5 ps^−1^(dashed curve), and 16 ps^−1^(dotted curve), respectively. (**b**) Transmittances of graphene on the grating with the periods of Λ = 3 μm(solid curves), 5 μm(dashed curve), and 10 μm(dotted curve), respectively. The scattering rate is assumed *γ* = 16 ps^−1^, and the gap size *w* = Λ/30 is proportional to the size of the period. The curve in the upper panel assumes zero chemical potential of graphene. The frequency *ω* indicates the locations of transmittance minimum.

The graphene has been assumed in good quality with a scattering of *γ* = 1 ps^−1^ for the data shown in the above figures. The resonance frequency *ω* of graphene SPPs, found by using Eq. (1), is at the transmittance minimum in a sharp valley, as shown in Fig. 2a in the graphene SPP dominant region. The transmittance curve for *d*_1_ = *d*_2_ = 10 nm in Fig. 2a is also plotted in Fig. 4a as the solid curve. In our case where *Kd*_2_ ≪ 1, the resonance frequency varies as *ω* ∝ Λ^−1^ according to Eq. (1).

In the case of a high scattering rate, the resonances of graphene SPPs become broad; eventually the individual resonances cannot be distinguished for a sufficiently high scattering rate. This phenomenon is shown in Fig. 4a, where the graphene sheet is assumed to have a scattering rate of *γ* = 1 ps^−1^, 5 ps^−1^, and 16 ps^−1^, respectively, with other simulation parameters being the same as those used for Fig. 2a. As can be seen, in the case of *γ* = 16 ps^−1^, the resonances become so broad that they merge and become a broad trough. Nevertheless, the absorption of graphene SPPs is still apparent, as can be seen by comparing the transmittance curve calculated using *μ* = 0 in the upper panel of Fig. 4b, and the transmittance curves calculated using *μ* = 100 meV in the lower panel of Fig. 4b. If we regard the transmittance minimum as the new effective resonance frequency *ω*, we can still observe the trend that shows the increasing *ω* with decreasing Λ, approximately following the relation *ω* ∝ Λ^−1^. This trend is shown in Fig. 4b, where the transmittance curves are calculated using Λ = 3 *μ*m, 5 *μ*m, and 10 *μ*m, respectively. The increasing *ω* with decreasing Λ is due to the fact that the broad transmittance trough is the collective result of the many resonances given by Eq. (1), which dictates the relation that *ω* ∝ Λ^−1^.

### Experimental studies

**Figure 5:**
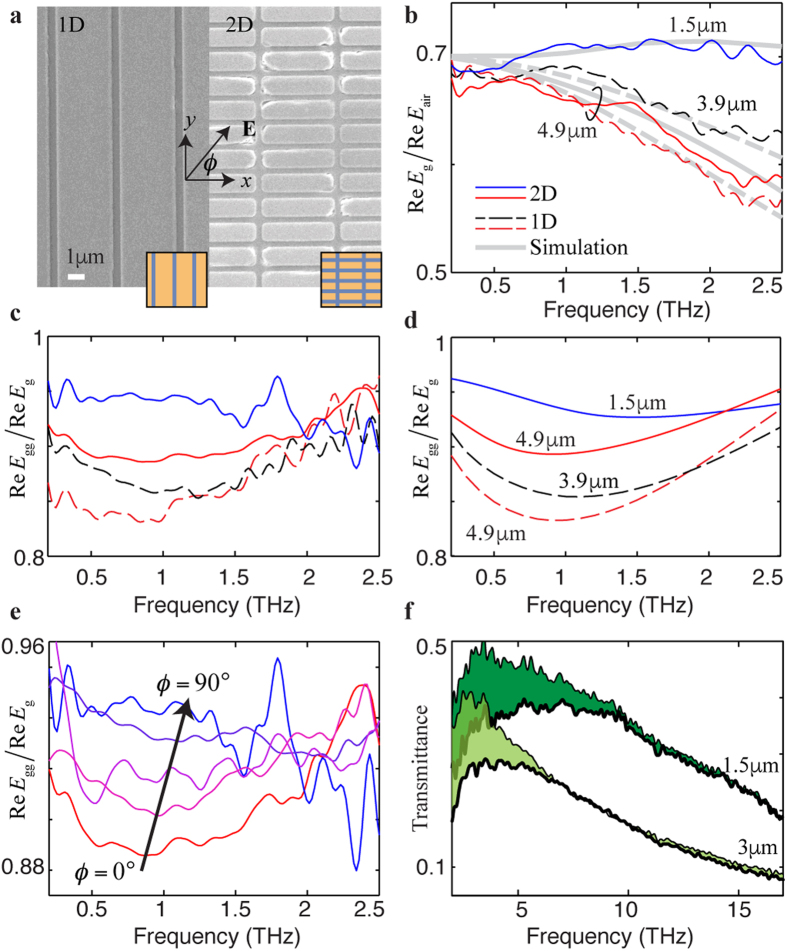
THz-TDS and FTIR measurements of the graphene-grating structures. (**a**) Images of scanning electron microscope for 1D and 2D gratings. The 1D gratings shown in Fig. 1d are characterized by the parameters: *w* = 0.4 μm, *d*_1_ = 50 nm, *d*_2_=30 nm, 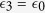, 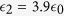 for silicon dioxide, 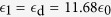 for silicon, Λ = 3.9 μm and 4.9 μm, respectively, for two samples. The 2D structure is the same as the 1D structures except that the 2D structure is also periodic in the *y* direction. The long and short periods are Λ = 4.9 μm and 1.5 μm, respectively. These metallic gratings are made of titanium nitride (TiN), represented by the yellow regions in the insets. (**b)** Ratio of the real part of the transmitted field through gratings, *E*_g_, to that of the free space without the grating, *E*_air_. For the 2D grating (solid curves), both *ϕ* = 0° and 90° for the polarization along the periods of Λ = 4.9 μm and 1.5 μm, respectively, are measured. For the 1D gratings (dashed curves), only *ϕ* = 0° is measured. Theoretical curves, plotted as grey curves, are calculated using the classical modal method^15^ for the 1D structures, and using COMSOL software for the 2D structure. A conductivity of *σ* = 6000 Ω^−1^cm^−1^ is taken for TiN^24^. (**c)** Ratio of the measured real part of the transmitted field through graphene-grating structure, *E*_gg_, to the real part of *E*_g_. (**d)** Simulation results for (**c)** The fitting parameters are *μ* = 60 meV and *γ* = 16 ps^−1^ except for the period Λ = 4.9 μm of the 2D grating, which is fitted using the scattering rate of *γ* = 29 ps^−1^. (**e**) Re*E*_gg_/Re*E*_g_ for different values of the polarization angle *ϕ* = 0°, 35°, 45°, 65°, and 90°. The arrow indicates the direction of increasing angles. (**f**) Transmittance measurement of FTIR for 1D gratings with (thick curves) and without (thin curves) graphene on top; the green areas show the transmittance difference. Two different samples of periods Λ = 3 μm and 1.5 μm are measured.

To demonstrate the concept device based on Eq. (1) as well as the simulation results shown in Fig. 4, we fabricate a structure shown in Fig. 1d. 1D and 2D grating structures of different periods Λ are fabricated with other structural parameters kept unchanged for all structures (see method), as shown in Fig. 5a. Terahertz time-domain spectroscopy (THz-TDS)^20^ is used to measure the transmitted electric field through the grating, 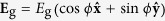, and through the free space without the grating, ***E***_air_, at room temperature, where *ϕ* is the azimuth angle of the incident light (see method). The values of the ratio Re*E*_g_/Re*E*_air_ are plotted as dotted curves in Fig. 5b for two different 1D gratings that have periods of Λ = 3.9 *μ*m and Λ = 4.9 μm, respectively. For the 2D grating, the values of the transmitted electric field ratio Re*E*_g_/Re*E*_air_ for the incident THz fields polarized at the angle of *ϕ* = 0° along the long period of Λ = 4.9 μm and at the angle of *ϕ* = 90° along the short period of Λ = 1.5 *μ*m, respectively, are plotted as solid curves in Fig. 5b. The agreement between the experimental data and the simulation results shows the good quality of our grating structures.

Monolayer graphene grown by chemical vapor deposition (CVD) is then transferred onto the grating structures so that we have a system shown in Fig. 1d. The transmitted electric field of the graphene-grating system Re*E*_gg_ is divided by Re*E*_g_ so that the resonance locations of the graphene SPP mode can be observed. The experimental and simulation results are plotted in Fig. 5c,d, respectively. The simulation is carried out using the fitting parameters of *μ* = 60 meV and *γ* = 16 ps^−1^ except for the period Λ = 4.9 μm of the 2D grating, which is fitted using a scattering rate of *γ* = 29 ps^−1^. The high scattering rate might be due to the ripples caused by the uneven surface of the 2D grating structure, or due to angle-sensitive scattering centers such as grain boundaries that prefer to grow in a certain direction resulting in a high scattering rate at specific polarization angles. By rotating the angle from 0° to 90° for the 2D sample, as plotted in Fig. 5e, a smooth transition is shown for the curves of Re*E*_gg_/Re*E*_g_ at different angles. This further indicates the angle-dependent scattering rate and the polarization-sensitive nature of our 2D structure. It can also be seen that the resonance frequency of the SPP mode shifts to a higher frequency for a shorter grating period, which is consistent with the simulation results shown in Fig. 4b. The resonances shown in Fig. 5c,d are expected to become sharper if the scattering rate is reduced, as the simulation suggests in Fig. 4a. The scattering rate as low as 1 ps^−1^ is physically possible as has been already measured for a graphene sheet that has a mobility of 230,000 cm^−1^V^−1^s^−1^ and a carrier density of 2 × 10^11^ cm^−2^ 21.

The effect of Drude conductivity is observed using Fourier-transform infrared spectroscopy (FTIR). The transmittance of 1D gratings with periods of Λ = 1.5 μm and Λ = 3 μm are measured with and without the graphene layer. As can be seen in Fig. 5f, the differences in the transmittance, which are shown as the green areas, indicate the frequency region where the Drude conductivity is significant. This large Drude absorption of graphene is also shown in Fig. 4b. Because the graphene SPP mode is supported by the Drude conductivity, these differences shrink quickly as the frequency increases, indicating that the excitation of the graphene SPP mode at high frequencies gradually becomes difficult. Therefore, the operational range of the graphene SPP-based THz device shown in Fig. 1d is limited not only by intrinsic Landau damping^22^, but also by the frequency-dependent nature of the Drude conductivity of graphene.

## Discussion

We have shown the characteristic transmittance curves of various graphene-based THz devices utilizing the excitation and coupling of different surface modes. The dispersion curves of these modes are found through both simulation and analytical solutions. The coupling of graphene SPPs with the cavity modes is also discussed. The coupling results in the gap openings in the dispersion curves of the cavity modes. The locations of the gaps can be tuned by tuning the chemical potential of graphene, and therefore can be utilized in a graphene-based modulator.

In the case of a very high scattering rate, the individual resonances of graphene SPPs become so broad that they merge and become one broad trough with a new effective resonance frequency *ω*. The effective *ω* approximately follows the relation *ω* ∝ Λ^−1^ due to the fact that the broad transmittance trough is the collective result of the resonances given by the relation *ω* ∝ Λ^−1^. The effective *ω* of structures with different periods are experimentally measured in a THz-TDS experiment. As predicted, the effective *ω* increases with decreasing grating period. The angle-dependent scattering rate and the polarization-sensitive nature of the 2D structure are shown by rotating the polarization direction of the incident THz wave. It is also shown that the excitation phenomenon gradually disappears at high frequencies due to the decreasing Drude conductivity of graphene, consistent with the simulation results. The decrease of the Drude conductivity with increasing frequency imposes another limitation in the operational frequency range of a graphene-based THz device besides the losses of the SPP mode due to Landau damping at high frequencies.

## Methods

### Sample fabrication

Following the standard photolithography process, 50-nm deep trenches along the *y* direction were developed on a Si wafer. For the samples of 1D slit grating, the spacing between neighboring trenches was 0.4 μm, and the width was Λ–0.4 μm, where Λ was the period of the structure. Various periods for different 1D samples were developed on the Si wafer. For the samples of 2D slit grating, trenches along the *x* direction were also developed, as shown in the right panel of Fig. 5a. The Si wafer was then deposited with titanium nitride (TiN). Redundant TiN above the trenches was removed by chemical-mechanical planarization (CMP) so that the surface of the wafer was smooth with TiN embedded in the Si wafer. Subsequently, a 30-nm thick silicon dioxide layer was deposited. The wafer was then cut into 1D and 2D samples of different periods. Finally, monolayer graphene grown by CVD is transferred on top of each sample.

### THz-TDS measurement

The generation and detection of THz pulsed radiation was set up on a mode-locked Ti:sapphire laser operating at 800-nm wavelength with 42-fs pulses at a 75-MHz repetition rate. The femtosecond-pulse laser beam was split into a pump beam and a probe beam. The THz pulses were generated by using the pump laser pulses to trigger a low-temperature-grown GaAs photoconductive dipole antennas characterized by a wire distance of 20 μm and a gap size of 5 μm. The generated THz-pulse beam was focused by a pair of off-axis parabolic mirrors onto a sample at normal incidence so that the polarization is parallel to the sample surface. The angle *ϕ* of the polarization is varied by carefully rotating the sample. The temporal electric-field-amplitude profile of the transmitted THz pulse was sampled by scanning the delay between the pump and probe optical pulses. For this sampling measurement, the transmitted THz pulse being measured was focused by another pair of off-axis parabolic mirrors on an antenna of the same type as that for the THz generation. In order to avoid any undesirable effects caused by the humidity in the laboratory air, the THz-TDS system was placed in a vacuumed Plexiglas box. The collected time-domain data was then Fourier transformed to the frequency-domain data.

Because the metallic grating also works as a polarizer, only the incident field along the grating direction at *ϕ* = 0° was considered for the 1D structures. The transmitted electric field for the polarization at *ϕ* = 90° was too small, and the signal-to-noise ratio was too low for any meaningful data, indicating negligible depolarization through the grating. The value of 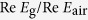 was calculated to obtain the true response of the grating and to eliminate the influence from air and other optical components. For each polarization angle *ϕ*, *E*_g_ and *E*_air_ are measured separately. The same procedure was also taken for the measurements of *E*_g_ and *E*_gg_.

## Additional Information

**How to cite this article**: Lin, I.-T. *et al*. Family of graphene-assisted resonant surface optical excitations for terahertz devices. *Sci. Rep.* **6**, 35467; doi: 10.1038/srep35467 (2016).

## Supplementary Material

Supplementary Information
