# Supplementary material for: Family of graphene-assisted resonant surface optical excitations for terahertz devices
Source: Sci Rep. 2016 Oct 14;6:35467. doi: 10.1038/srep35467 (PMC5064388; doi:10.1038/srep35467)
Supplement: Supplementary Information [file srep35467-s1.pdf]

Family of graphene-assisted resonant surface optical excitations for terahertz devices

Supplementary Information

I-Tan Lin[1], Jia-Ming Liu[1*], Hsin-Cheng Tsai[2], Kaung-Hsiung Wu[2*], Jheng-Yuan Syu[3], Ching-Yuan Su[3]
[1]Electrical Engineering Department, University of California, Los Angeles, Los Angeles, California 90095, USA.
[2]Department of Electrophysics, National Chiao-Tung University, Hsinchu, Taiwan
[3]Graduate Institute of Energy Engineering, National Central University, Taoyuan, Taiwan
*Corresponding authors: liu@seas.ucla.edu

For a graphene sheet located at a distance of d_2 above a perfectly conducting metal, the characteristic dispersion of the graphene SPP mode is obtained by solving the equation

$$\epsilon_2 + \left(\epsilon_3 + \mathrm{i}\beta\frac{\sigma}{\omega}\right)\tanh\beta d_2 = 0, \quad \text{(S1)}$$

where as defined in the main text, ϵ_2 and ϵ_3 are the permittivities below and above the graphene sheet, respectively; σ is the conductivity of graphene; and β is the propagation constant of the SPP mode. If the Drude model is used for the graphene conductivity and the carrier scattering rate γ is ignored, σ is imaginary and β has to be real for a solution to exist. Furthermore, if the periodic boundary condition is imposed, β is given by $\beta = 2m\pi/\Lambda$, where m is a positive integer and Λ is the period. Then, we retrieve Eq. (1) in the main text from Eq. (S1).

When the scattering rate γ is not negligible, the imaginary part of the propagation constant β becomes important, and Eq. (S1) has to be used instead of Eq. (1). The simulated values of $\mathrm{Re}\,E_{\mathrm{gg}}/\mathrm{Re}\,E_{\mathrm{g}}$ calculated using the classical modal method are plotted in Fig. S1 for scattering rates of $\gamma = 0.1$, 1, 3, and $16\,\mathrm{ps}^{-1}$, respectively. Because of the finite values of γ, Eq. (S1) has a solution only if β is complex: $\beta = \beta' + \mathrm{i}\beta''$ with β' and β'' having real values. As can be seen in Fig. S1, four resonances are observed for $\gamma = 0.1\,\mathrm{ps}^{-1}$, which from low frequency to high frequency corresponds to the first- to fourth-order modes of the graphene SPP, respectively. As the scattering rate increases, both the broadening and the shift of the resonances are observed. The redshift is most significant for the highest-order mode. For example, for the third order, $\beta' = 3\cdot 2\pi/\Lambda$, we find from Eq. (S1) that $(\beta'', \nu) = (0.033\pi/\Lambda, 1.486\,\mathrm{THz})$ for $\gamma = 0.1\ \mathrm{ps}^{-1}$, $(\beta'', \nu) = (0.237\pi/\Lambda, 1.484\,\mathrm{THz})$ for $\gamma = 1\ \mathrm{ps}^{-1}$, and $(\beta'', \nu) = (0.97\pi/\Lambda, 1.468\,\mathrm{THz})$ for

$\gamma = 3\ \text{ps}^{-1}$. For $\gamma = 16\ \text{ps}^{-1}$, the linewidths of the resonances are so broad that the four resonance frequencies are not distinguishable. Nevertheless, as discussed in the main text, if we regard the transmittance minimum as the effective resonance frequency ν, we can still observe the trend that shows the increasing ν with decreasing Λ, approximately following the relation $\nu \propto \Lambda^{-1}$.

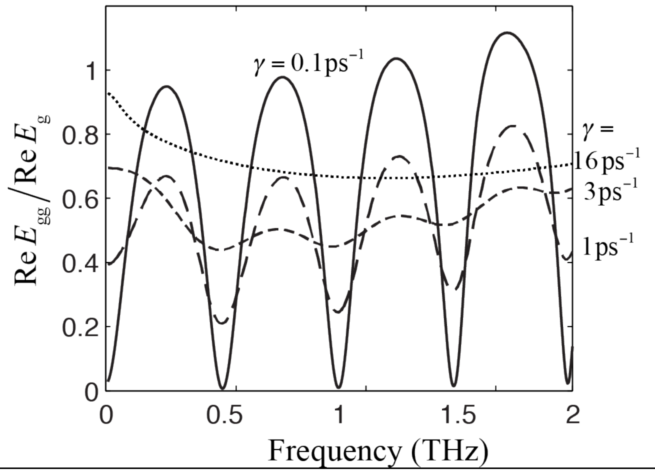

Figure S1 | $\text{Re}\,E_{gg}/\text{Re}\,E_g$ **for graphene of different scattering rates.** The simulation parameters are the same as those used in Fig. 3 described in the main text except that here the metal is assumed to be a perfect conductor. The period is $\Lambda = 4.9\ \mu\text{m}$.
